# Cellular Notch responsiveness is defined by phosphoinositide 3-kinase-dependent signals

**DOI:** 10.1186/1471-2121-7-10

**Published:** 2006-02-28

**Authors:** Grahame Mckenzie, George Ward, Yvette Stallwood, Emmanuel Briend, Sofia Papadia, Andrew Lennard, Martin Turner, Brian Champion, Giles E Hardingham

**Affiliations:** 1 Lorantis Ltd., 410 Cambridge Science Park, Cambridge, CB4 0PE, UK; 2 Centre for Neuroscience Research, University of Edinburgh, Summerhall, Edinburgh, EH9 1QH, UK; 3 The Laboratory of Lymphocyte Signalling and Development, The Babraham Institute, Cambridge, CB2 4AT, UK; 4 Inion Ltd, Unit 9B, Cambridge Science Park, Cambridge, CB4 0FG, UK

## Abstract

**Background:**

Notch plays a wide-ranging role in controlling cell fate, differentiation and development. The PI3K-Akt pathway is a similarly conserved signalling pathway which regulates processes such as differentiation, proliferation and survival. Mice with disrupted Notch and PI3K signalling show phenotypic similarities during haematopoietic cell development, suggesting functional interaction between these pathways.

**Results:**

We show that cellular responsiveness to Notch signals depends on the activity of the PI3K-Akt pathway in cells as diverse as CHO cells, primary T-cells and hippocampal neurons. Induction of the endogenous PI3K-Akt pathway in CHO cells (by the insulin pathway), in T-cells (via TCR activation) or in neurons (via TrKB activation) potentiates Notch-dependent responses. We propose that the PI3K-Akt pathway exerts its influence on Notch primarily via inhibition of GSK3-beta, a kinase known to phosphorylate and regulate Notch signals.

**Conclusion:**

The PI3K-Akt pathway acts as a "gain control" for Notch signal responses. Since physiological levels of intracellular Notch are often low, coincidence with PI3K-activation may be crucial for induction of Notch-dependent responses.

## Background

The phosphoinositide-3-kinases (PI3Ks) constitute an enzyme family of p85/p110 heterodimers responsible for regulating numerous biological processes in diverse cell types [1] such as cellular proliferation, differentiation and survival. Of particular interest is the role of this pathway in lymphocyte activation, where antigen and cytokine receptor ligation result in activation of PI3K-driven signals [2]. A key target of PI3K activity is Akt/PKB, which mediates many of the effects of PI3K activation [3].

PI3K is activated in T-cells both in response to T-cell receptor (TCR) stimulation, and by engagement of the co-stimulatory receptor CD28. Gene targeting studies have emphasised the essential role PI3K plays in these responses: whereas PI3K-deficient mice are immunodeficient [4,5], constitutive activity is associated with hyperproliferative T-cell responses and autoimmunity [6]. Equally, preventing the termination of PI3K activity by conditional gene targeting of the lipid phosphatase *Pten *in T-cells leads to autoimmunity and development of lymphoma [7]. These studies demonstrate that PI3K activity is tightly regulated and plays an integral role in the maintenance of lymphocyte homeostasis.

The Notch signalling pathway plays a critical role in a wide range of developmental processes in the embryo, including binary lineage decisions and boundary formation [8]. Binding of the Notch ligands Delta or Jagged to the Notch receptor instigates proteolytic cleavage which releases the Notch intracellular domain (N-IC) [8] which activates the transcription factor CBF-1 which, in turn, controls target genes such as Hes-1 [9]. Central to Notch's role is its ability to modulate the differentiative potential of pluripotent cells, for instance in the neural stem cell compartment. The effects of Notch also extend to the developing immune system, where it directs the differentiation of lymphoid precursors [10]. Recent data have highlighted unexpected functions for the Notch pathway in regulating cells of the peripheral immune system. Co-incident delivery of Notch signals to T-cells exerts a profound effect upon the outcome of antigen-stimulation *in vitro *[11-13] suggesting that interplay between the antigen receptor and Notch pathways regulate the outcome of peripheral T-cell responses.

Mice expressing catalytically inactive PI3K subunit p110δ [5] or a deletion of p110δ [4] exhibit defective B-cell and T-cell receptor signalling: antigen-mediated activation of BCR or TCR fails to activate Akt and cells exhibit defective proliferation and cytokine responses. Recent publications have demonstrated that Notch signals modulate cytokine production by T-cells [12,13], suggesting that the Notch pathway integrates with TCR-mediated signals to modulate cytokine production. Furthermore, marginal zone B (MZB) cell development is severely impaired in both p110δ- and Notch2-deficient mice [4,14]. Taken together with Notch's ability to modulate TCR signals, this phenotypic similarity suggested to us the possibility of Notch/PI3K signalling integration. Our studies reveal that the PI3K-Akt pathway is a potent inducer of Notch-dependent responses in cell types as diverse T-cells and neurons, demonstrating that this is a cell signalling paradigm with broad applicability.

## Results

### Inhibition of the PI3K-Akt pathway impairs Notch signalling in Jurkat T-cells

**Figure 1:**
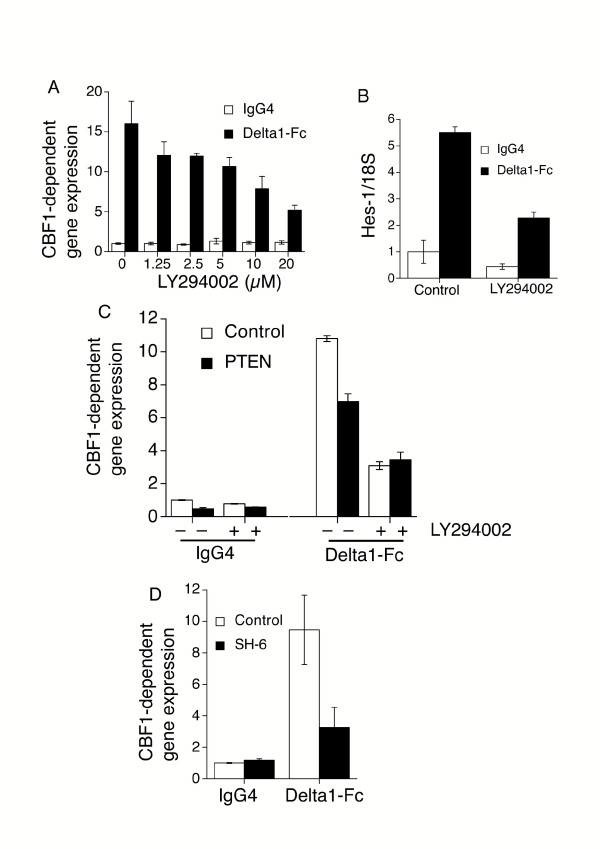
**Inhibition of the PI3K-Akt pathway impairs Notch signalling in Jurkat T-cells**. **(A) **J-N2 cells were transfected with 14 μg of p10xCBF1-luc and 1 μg pTK-RL, incubated overnight, then plated onto 96-well plates coated with either 20 μg/ml hIgG4 or 20 μg/ml hDelta1-Fc along with LY294002 for 6 hours. CBF1-reporter gene activity is normalised to Renilla control in this and all luciferase assays. **(B) **J-N2 cells were plated onto either hIgG4 or Delta1-Fc ± 10 μM LY294002, and incubated for 8 hours before being processed for Hes1 mRNA levels by Q-PCR (Hes1/18 rRNA). **(C) **J-N2 cells were transfected with 10 μg of pCBF1x10-luc, 4 μg of Pten expression construct or empty pcDNA3.1 vector,1 μg of pTK-RL, and treated with either 20 μg/ml hIgG4 or 20 μg/ml hDelta1-Fc ± 10 μM LY294002. CBF1-reporter activity was measured after 6 hr. All data is presented as mean ± s.d., and is representative of three repeat experiments. **(D) **J-N2 cells were transfected with p10xCBF1-luc and pTK-RL and stimulated as described in Fig. 1A, except that cells were treated with Akt inhibitor SH-6 (50 μM) or DMSO vehicle for 6 hours.

To address whether the PI3K pathway is a regulator of Notch-responsiveness, we generated a Jurkat E6.1 T-cell line which stably expresses full-length human Notch2 cDNA (J-N2 cells). J-N2 cells were treated with plate-bound recombinant human Delta1 protein (hereafter referred to as Delta1-Fc) fusion protein, and Notch signalling was measured using a transiently-transfected CBF1-driven luciferase reporter construct (p10xCBF1-luc). Plate-bound recombinant Delta1-Fc efficiently transactivated CBF1-luc, driving around a 10-fold increase in activity (Fig. 1A). A similar result was obtained using the murine Hes-1 promoter cloned upstream of the luciferase reporter (data not shown). Strikingly, treatment of these cells with the PI3K inhibitor LY294002 led to a dose-dependent decrease in Delta1-Fc-induced Notch signalling (Fig. 1A). A similar result was observed with the mechanistically-distinct PI3K inhibitor Wortmannin (data not shown). The integrity of this reporter assay as an accurate reflection of Notch signalling was confirmed by assessing mRNA levels of the Notch target gene Hes-1 by Q-PCR in Delta1-Fc-treated J-N2 cells (Fig. 1B). As with the CBF1-dependent reporter, treatment with LY294002 inhibited the up-regulation of Hes-1 by Delta1-Fc.

The lipid phosphatase Pten dephosphorylates the 3' phosphoinositide products of PI3K catalysis and prevents downstream PI3K signaling [1]. Jurkat E6.1 cells are deficient in expression of Pten, and therefore have a highly active PI3K-Akt pathway [15]. We therefore hypothesised that reconstituting this enzyme would reduce Notch signalling. Transient expression of *Pten *in J-N2 cells led to a decrease in both basal and Notch ligand-induced activation of CBF1-dependent gene expression (Fig. 1C). *Pten*-transfected and control cells showed equivalent CBF1-reporter activity in the presence of LY294002, demonstrating that once PI3K activity is abolished pharmacologically, *Pten *over-expression becomes redundant.

The PI3K pathway activates several enzymes, including the central signalling molecule Akt/PKB. To further define the mechanism by which PI3K activity regulates Notch signals, we asked whether the inhibitory effect of LY294002 could be recapitulated with an inhibitor of Akt. Blocking Akt activity in J-N2 cells with the selective inhibitor SH-6 [16] inhibited Delta1-Fc-mediated upregulation of Notch signalling (Fig. 1D). Taken together, these data identify the PI3K-Akt pathway as being responsible for the high level of ligand-induced Notch activation in J-N2 cells.

### The PI3K-Akt pathway regulates Notch-responsiveness in primary human CD4^+ ^T-cells

**Figure 2:**
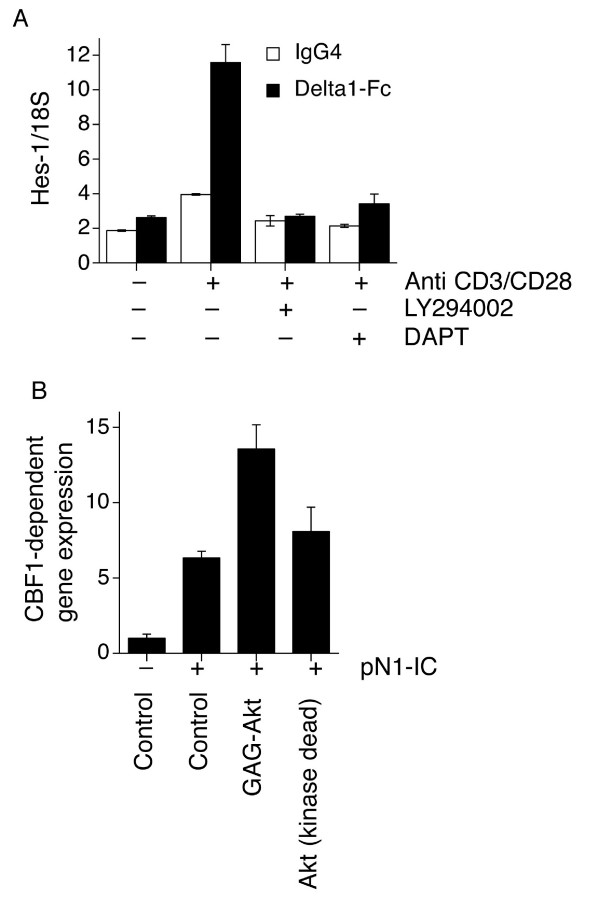
**The PI3K-Akt pathway regulates Notch-responsiveness in primary human CD4^+ ^T-cells**. **(A) **24-well tissue culture plates were coated with anti-human IgG4 and anti-mouse IgG2a capture antibodies at 1 μg/ml in PBS, and incubated at 4°C, overnight. Appropriate stimuli added: Delta1-Fc or hIgG4 as control, both at 20 μg/ml in PBS and anti-CD3 (Hit3a, IgG2a) or mIgG as control, both at 1 μg/ml in PBS, and incubated at 37°C, 3 h. Plates were washed with PBS and positively selected hCD4+ T-cells were seeded at 2 × 10^6 ^cells/well, in 1 ml of media to a total of 1 × 10^7 ^cells/stimulation. Cells were stimulated overnight ± 10 μM DAPT or 10 μM LY294002 and 2 μg/ml anti-CD28 where applicable. Cells were pooled, RNA prepared and cDNA synthesised as described in Methods. Relative Hes1 expression was determined by Q-PCR. Data shown represents triplicate assays of cDNA prepared from pooled cells, and is representative of two individual experiments performed using CD4+ T-cells prepared from different donors.**(B) **Human primary CD4^+ ^T-cells were nucleofected with 4 μg p10xCBF-luc, 0.5 μg pTK-RL and 1 μg of either pN1-IC or empty pcDNA3.1, and 2 μg of either gagAkt or kinase-dead (KD) Akt construct. CBF1-reporter activity is presented as mean normalised luminescence ± s.d. Data representative of two repeat experiments from different blood donors.

We next investigated whether PI3K activity also regulates Notch-responsiveness in primary human CD4^+ ^T-cells. We asked whether PI3K induction, triggered by TCR activation, could influence Hes-1 levels (Fig. 2A). In the absence of any exogenously added Delta 1 (open bars-IgG control), TCR activation (with anti-CD3/anti-CD28 antibodies) up-regulated Hes-1 levels in a LY294002-sensitive manner. Significantly, this induction was also blocked by the gamma secretase inhibitor DAPT, consistent with an absolute requirement for N-IC. We also studied the induction of Hes-1 by Delta 1-Fc treatment (closed bars). In the absence of TCR activation, Delta 1-Fc induced a small, but consistent induction of Hes-1. However, coincidence of Delta 1-Fc with TCR activation (anti-CD3/anti-CD28 antibodies) dramatically up-regulated Hes-1 levels in a LY294002-sensitive manner. Again, this induction was also blocked by the gamma secretase inhibitor DAPT. LY294002 also inhibited Notch signalling in primary human CD4^+ ^T-cells transfected with p10xCBF1-luc and a Notch1 intracellular domain expression plasmid (data not shown), demonstrating that the effect of PI3K on Notch is exerted downstream of Notch cleavage. Thus, TCR activation dramatically increases the cellular response to Notch signals in primary human T cells via a mechanism requiring the PI3K-Akt pathway.

We then tested whether Akt activation is sufficient to boost Notch-responsiveness in human T cells. Over-expression of a constitutively-active Akt construct (gagAkt [17]) was sufficient to potentiate the ability of pN1-IC to drive CBF1-dependent reporter activity (Fig. 2B).

### PI3K regulates Notch signals through GSK3β activity

**Figure 3:**
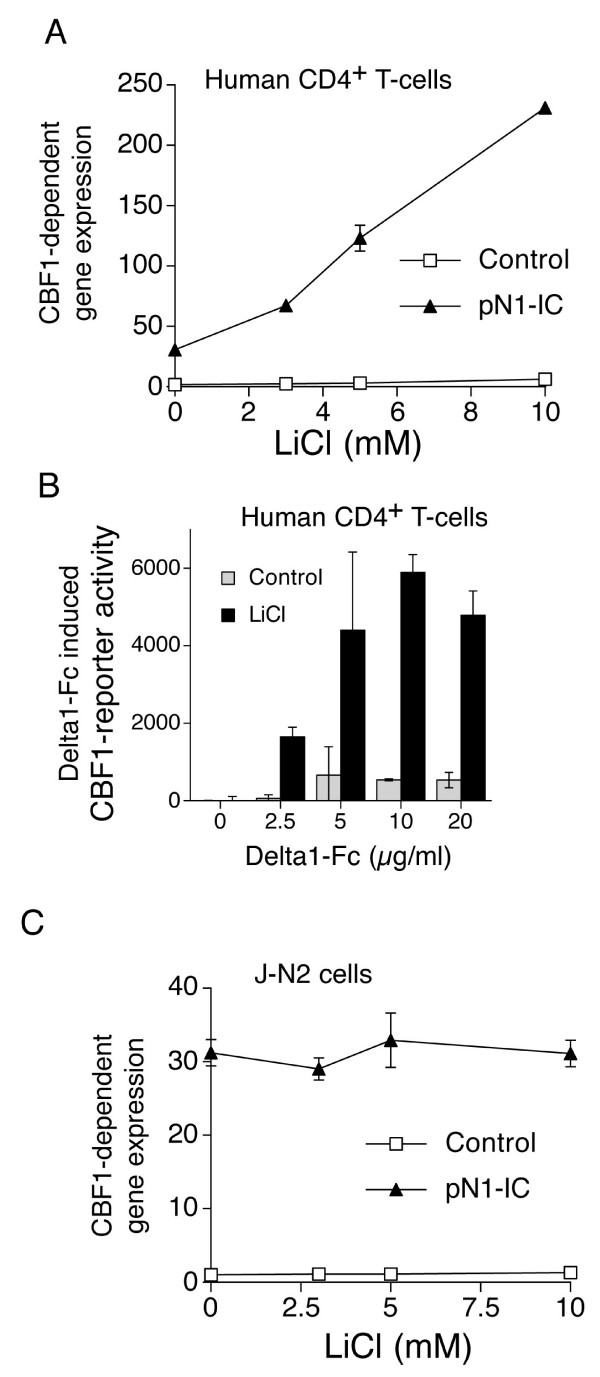
**Inhibiting GSK3β with lithium chloride enhances Notch signals in primary human CD4^+^, but not Jurkat, T-cells**. **(A) **Primary human CD4^+ ^T-cells were nucleofected with 4 μg p10xCBF-luc, 0.5 μg pTK-RL and 1 μg of either pN1-IC or pcDNA3.1 and plated o/n in triplicate with increasing concentrations of LiCl. Data is presented as the fold induction in normalised luminescence compared to the untreated vector only control. **(B) **Primary human CD4^+ ^T-cells were nucleofected with 4 μg p10xCBF-luc and exposed to different levels of plate-bound Delta1-Fc in the presence or absence of LiCl (10 mM). (C) J-N2 cells were transfected with p10xCBF1-luc and pTK-RL and stimulated overnight in increasing concentrations of LiCl. Data is presented as the fold induction in normalised luminescence compared to the untreated IgG4 control.

Previous data has suggested that inactivation of GSK3β by the Wingless pathway enhanced Notch signals by preventing inhibitory phosphorylation of N-IC by GSK3β [18]. Therefore, given that Akt phoshorylates and inactivates GSK3β [19], we hypothesised that Akt-dependent inactivation of GSK3β might be responsible for Notch potentiation. To address this question, we looked at the N1-IC-induced Notch response in primary human CD4^+ ^T-cells treated with the GSK3β inhibitor lithium chloride. Increasing concentrations of LiCl induced a dramatic increase in Notch signalling (Fig. 3A).

In contrast to N1-IC overexpression, triggering Notch signalling using recombinant ligand to ligate endogenous full length Notch generates far weaker signalling. This fact, coupled with limited transfection efficiency of primary T cells, makes it technically difficult to achieve robust induction of a transfected CBF1 reporter construct by Delta1-Fc. However, given the large potentiation of Notch-responsiveness by LiCl (Fig. 3A), we hypothesised that, in the presence of LiCl, robust Delta1-Fc dependent induction of a CBF1 reporter could be observed. We found that, consistent with the technical issues outline above, plate-bound Delta1-Fc activates the CBF1 reporter weakly and unreliably in the absence of LiCl (Fig. 3B). However, LiCl greatly potentiates activation of the reporter by plate-bound Delta1-Fc (Fig. 3B). Thus, the GSK3β inhibitor LiCl is sufficient to potentiate primary Notch-responsiveness in primary human CD4^+ ^T-cells, triggered either by N1-IC expression, or by ligation of endogenous full length Notch by plate-bound ligand.

In Jurkat T-cells, deficient in *Pten *expression, basal Akt activity is high [15], so we expected GSK3β to be maximally inhibited. Consistent with this, LiCl treatment did not exert any further effect on Delta1-induced signalling in Jurkat-N2 cells (Fig. 3C).

### PI3K activity also regulates Notch responsiveness in CHO cells and neurons

**Figure 4:**
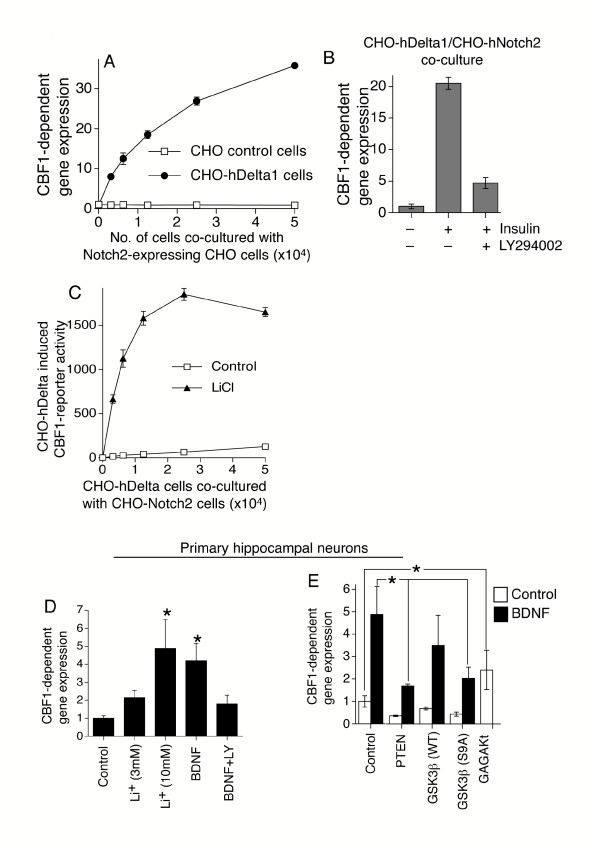
**The PI3k/GSK3β pathway also regulates Notch signalling in CHO cells and in primary neurons**. **(A) **CHO-N2 cells were co-cultured with increasing numbers of either CHO-hDelta1 or control CHO cells and luciferase reporter activity assayed for after 24 hours. CBF1-dependent reporter activity is presented as the fold induction in luminescence versus CHO-N2 cell only value. Mean ± sem given. **(B) **CHO-N2 cells were co-cultured with 10^4 ^CHO-hDelta1 cells per well ± insulin ± LY294002 as indicated. CBF1-reporter activity is presented as fold induction of non-insulin stimulated cells. **(C) **CHO-N2 cells were co-cultured with increasing numbers of CHO-hDelta1 in the presence or absence of LiCl (10 mM). (**D**) Rat primary hippocampal neurons were transfected with 0.5 μg of p10xCBF1-luc and 0.05 μg pTK-RL, then stimulated overnight with either LiCl or 25 ng/ml BDNF ± 10 μM LY294002. **(E) **Neurons were transfected with 0.2 μg of p10xCBF1-luc, 0.05 μg pTK-RL, 0.05 μg of pN1-IC and 0.4 μg of the relevant expression plasmid. CBF1-reporter activity is presented as normalised luminescence ± s.e.m (n = 3).(*p < 0.05, Mann Whitney U test).

To investigate whether PI3K-dependent regulation of Notch responsiveness may be a general phenomenon extending beyond T-cell biology, we took advantage of a Notch-dependent CHO cell reporter assay which we have previously described [20]. In this assay, CHO cells which stably express a human Notch2 receptor cDNA and a CBF1-dependent luciferase reporter construct (termed CHO-N2) are co-cultured with a second CHO cell line which stably expresses the full-length human Delta1 cDNA (termed CHO-hDelta1). CHO-hDelta1 cells trigger a dose-dependent luciferase response to the CHO-N2 cells, whilst control CHO parental cells fail to activate the Notch pathway (Fig. 4A). We next asked if activation of the PI3K pathway influenced Notch signals in this assay system. As predicted from our T-cell studies, including insulin in the culture medium potentiated Notch signalling, and this effect was blocked almost completely by inclusion of LY294002 (Fig. 4B). From our observations with T-cells, we hypothesisesd that GSK3β inhibition with LiCl could substitute for insulin in potentiating Notch signalling induced by CHO-hDelta1 cells. LiCl does indeed potentiate Notch signalling in the CHO co-culture system, leading to a substantial enhancement of ligand-dependent reporter activity (Fig. 4C).

**Figure 5:**
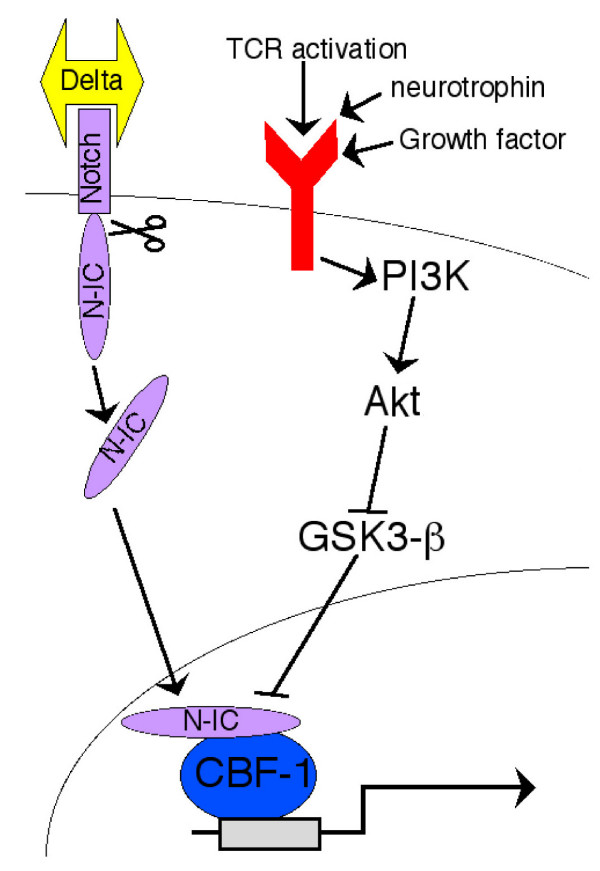
**A schematic depicting a proposed mechanism for the influence of the PI3K pathway on Notch-responsiveness**. See text for discussion of this model.

We then extended our study further, this time into primary neurons, cell types where Notch plays an emerging role in processes such as dendritic patterning and synaptic plasticity [21,22]. As observed with T-cells, inhibition of GSK3β with lithium chloride strongly enhanced the level of Notch signalling in rat primary hippocampal neurons in a dose-dependent manner (Fig. 4D). PI3K-activating neurotrophins such as BDNF regulate cell survival, proliferation and differentiation of both peripheral and central neurons [23]. Strikingly, BDNF treatment potentiated pN1-IC-driven reporter activity, and this was abrogated by treatment with LY294002 (Fig. 4D). *Pten *transfection ablated both basal and BDNF-stimulated reporter signalling, further underlining the role of the PI3K pathway in promoting Notch-responsiveness in neurons. Moreover, over-expression of constitutively active gagAkt was sufficient to potentiate pN1-IC-driven reporter activity (Fig. 4E). To further reinforce this finding, expression of a constitutively-active GSK3β construct (S9A), which is resistant to inactivation by Akt, prevented BDNF induction of Notch responsiveness (Fig. 4E). In contrast, expression of wild-type GSK3β, which is inhibited by Akt activity, failed to significantly reduce reporter activity. As observed with T-cells, inhibition of GSK3β with lithium chloride strongly enhanced the level of Notch signalling in a dose-dependent manner (Fig. 4D). Thus the PI3K-Akt pathway positively regulates Notch signals via GSK3β in neurons as well as T-cells, as summarised in Fig. 5.

## Discussion

The dynamic and interactive nature of intracellular communication dictates that cells are in receipt of multiple environmental signals at any given time. The cellular output must represent a net response to a diverse set of cues, and therefore the pathways mediating these responses are frequently integrated. Our studies demonstrate for the first time that PI3K activity strongly potentiates Notch responsiveness in diverse cell types. Given the vital nature of PI3K and Notch in controlling cellular survival and differentiation, the implications of this finding are far-reaching.

### Reciprocal signalling between the Notch and PI3K pathways in T cells

Ligation of lymphocyte antigen receptors leads to activation of PI3K, initiating a diverse and wide-ranging set of signalling pathways [2]. Our findings that PI3K activation enhances Notch signalling leads us to suggest that Notch pathway activation might be an inherent consequence of lymphocyte activation. This positions the Notch pathway as a central mediator of antigen-dependent stimulation of immune cells.

T-cells are entirely quiescent in the absence of antigen signals, but are shifted rapidly into a state of activation following TCR ligation. Inappropriate control of this activation can lead to autoimmune disease, and therefore negative feedback loops are an inherent feature of T-cell biology. Recent data show that Notch signals block Akt activation in peripheral CD4+ T-cells [13]. Indeed we also observe that in primary human CD4+ T-cells recombinant Delta1-IgG4 fusion protein, or Notch-IC expression, blocks TCR-mediated phosphorylation of Akt and GSK3β (GM, BC, unpublished data). Although Akt activation is essential for production of cytokines such as IL2 and IFNγ by T-cells [24], constitutive activity is associated with cellular transformation [25], and therefore the existence of a negative feedback loop to limit antigen-dependent Akt activation would appear rational. We have shown that co-incident Notch signals are enhanced upon TCR activation, and we hypothesise that by inhibiting Akt activity, enhanced Notch signalling may constitute a negative feedback control impacting upon peripheral T-cell activation.

It is interesting to note that whilst the model we propose is consistent with the data generated in peripheral T-cells, a number of studies suggest that the opposite is true [26-28]. Ciofani et al demonstrate that during the earliest stages of T-cell development in the thymus Notch signals co-operate with pre-TCR signalling to *activate *Akt-dependent metabolism, thereby promoting expansion of those T-cells which have undergone favourable TCR rearrangement. The functional outcome of PI3K/Notch pathway interaction thus appears to reverse from developing to mature T-cells, an observation which further underlines the ability of the Notch pathway to perform multiple, context-dependent, tasks during development. We suggest that a molecular understanding of how Notch can function as a bi-directional regulator of Akt activity is a research priority.

### PI3K and Wnt signalling modulate Notch responses via a common pathway

Our data strongly suggests that PI3K activity links to the Notch signalling pathway through Akt-mediated inactivation of GSK3β, a known downstream effector of Akt. In support of this data, PI3K and GSK3β [18,28,29] have both been identified as binding partners for N-IC in immunoprecipitation studies. GSK3β has also previously been positioned as a component of the downstream Notch signalling pathway in lateral inhibition in Drosophila [30]. Furthermore, GSK3β activity is inhibited downstream of both the wingless and PI3K pathways, and thus represents a convergent site for integration of extracellular signals with the Notch pathway. Indeed, inhibiting GSK3β activity by activating the wingless pathway with Wnt-1 has been shown to enhance Notch-dependent reporter activity in NIH-3T3 cells [18]. We note, however, that although an interaction between GSK3β and Notch seems conclusively proven, the functional consequences of this interaction are not consistent between different studies. Whilst our data agrees with that of Espinosa et al. [18], that is that GSK3β activity inhibits Notch signalling, data from the Nye laboratory in GSK3β-deficient murine embryonic fibroblasts demonstrates that GSK3β phosphorylates and stabilises Notch-IC, leading to enhanced signalling [29]. Although we have no definitive explanation for these experimental differences, it is possible that, as discussed above for thymocytes and T-cells, the effect of GSK3b on Notch changes between embryonic and mature tissues.

### Notch signalling in neural cells

Notch's role during embryonic development is not to directly program neuronal differentiation itself, but to inhibit this cell fate choice in neighbouring cells during the lateral inhibition process [8]. During neurogenesis, this is achieved by Notch target genes blocking transcription of pro-neural transcription factors such as MASH-1. Interestingly, conditional deletion of Pten in neural progenitors leads to dramatic alterations in brain architecture, an observation which the authors attribute to increased proliferation and self-renewal of neural stem cell populations [31]. Given that the data presented here establishes the Notch-suppressing capability of Pten, deregulated renewal of progenitors in these mice may be in part due to enhanced Notch signalling. This hypothesis awaits testing.

Notch also plays an emerging role in differentiating neurons by inhibiting dendritic outgrowth [21]. It is tempting to speculate that Notch-dependent dendritic retraction, reliant on neuron-neuron interactions, is augmented by target-derived neurotrophic factors promoting PI3K signalling. In differentiated neurons, Notch contributes to synaptic plasticity, learning and memory [22,32]. The long-standing involvement of BDNF and other neurotrophins in synaptic plasticity raise the possibility that PI3K-Notch interplay is at work in this scenario as well.

## Conclusion

Our data demonstrates that the potentiation of Notch signalling by PI3K is conserved in ontologically-distinct cell types, leading us to suggest that it represents a general mechanism of signal integration linking two evolutionarily-conserved pathways together. This apparently simple observation has far-reaching implications regarding the mechanisms by which stem cell self-renewal and differentiation proceeds. It will be interesting to assess the relevance of this finding in other signalling contexts, such as tumour formation, where Notch and PI3K have important roles.

## Methods

### Materials and plasmids

SH-6, lithium chloride, LY294002, Wortmannin and BDNF were from Calbiochem. Human IgG4 was from Sigma. Dual-Glo Luciferase assay system was from Promega. pN1-IC was constructed by inserting nucleotides coding for the intracellular domain of human Notch1 (amino acids 1758 to 2557) preceded by an initiator ATG codon into the BamHI and EcoRI sites of pcDNA3.1 (Invitrogen). pAdTATA-luc was constructed by inserting the Adenoviral major late promoter TATA sequence immediately 5' of the luciferase gene in plasmid pGL3Basic (Promega). p10xCBF1-luc was constructed by inserting 10 copies of the CBF1 binding site 5' of the AdTATA box in pAdTATA-luc. pTK-Renilla was from Promega. Human *Pten *expression construct was generated by PCR amplification of full-length human *Pten *from CD4^+ ^T-cell cDNA, and then cloned into the HindIII and EcoRI sites of pcDNA3.1. Constitutively-active (gagAkt) and kinase dead (Akt-KD) Akt expression constructs were a kind gift from Dr. Julian Downward [17]. Wild-type (WT) and constitutively-active (S9A) GSK3β constructs were a kind gift from Dr. J. Sadoshima [33]. All plasmids were prepared using the EndoFree maxiprep system (Qiagen).

### Cell culture

Jurkat E6.1 cells and Jurkat-N2 cells were grown in RPMI1640 (+10% v/v FBS, 2 mM L-glutamine). Primary CD4^+ ^T cells were isolated from freshly-collected donor blood as follows. Peripheral blood mononuclear cells were prepared by centrifugation over Ficoll-Paque, incubated with anti-CD4-coated magnetic beads (Miltenyi Biotec) and selected on LS positive selection columns (Miltenyi Biotec). After purification, primary CD4+ T-cells were cultured in RPMI1640 (supplemented with 10% v/v FBS, 2 mM L-glutamine and 5 × 10^-6^M β-mercaptoethanol). CHO-N2 and CHO-Delta1 cells have been described previously [20] and were routinely cultured in DMEM (+10% v/v/FBS, 2 mM L-glutamine). Rat primary hippocampal neurons were isolated and cultured as described [34], except growth media was supplemented with B27 (Gibco/BRL).

### Transfections and reporter assays

Jurkat E6.1 cells and Jurkat-N2 cells were transfected using Lipofectamine 2000 (Invitrogen). Cells were left 24 h post-transfection, washed, counted and re-plated in triplicate at 2 × 10^5 ^cells/well into flat-bottomed 96-well plates which had been (where relevant) pre-coated o/n at 4°C with hDelta1-Fc. Cells were stimulated, incubated o/n and luciferase activity measured using the Dual-Glo system (Promega). Firefly luciferase signal from the Notch-dependent p10xCBF1-luc was normalised using the Renilla luciferase signal driven by the pTK-Renilla.

Primary CD4^+ ^T cells were transfected using the Amaxa 'nucleofection' system according to the manufacturer's instructions. Briefly, 5 × 10^6 ^purified CD4^+ ^T-cells were resuspended in 100 μl of the appropriate Amaxa solution and transfected with 5 μg plasmid DNA using Protocol U-14. Cells were immediately transferred to 1 ml of pre-warmed media, counted and plated as required. After stimulation luciferase activity was detected using the Dual-glo assay system (Promega).

For neuronal experiments, rat primary hippocampal neurons were transfected after 9 days in culture using Lipofectamine 2000 and stimulated 36-48 hours later. Dual-Glo Luciferase assays were performed following o/n stimulation.

### Generation of Delta fusion protein (Delta1-Fc)

A fusion protein comprising the extracellular domain of human Delta1 fused to the Fc domain of human IgG4 was prepared by inserting a nucleotide sequence coding for the extracellular domain of human Delta1 (amino acids 1 to 537) into the expression vector pCON^© ^(Lonza Biologics). This plasmid was expressed in CHO cells, purified by affinity chromatography on ProteinA-Sepharose (Amersham Biosciences) before concentration and buffer exchange into PBS. Batch activity of this protein was confirmed as previously described [35].

### Delta1-Fc capture and immobilisation onto tissue culture plastic

To assess the effect of isolated Delta1-Fc signalling on Jurkat-N2 and CD4+ T-cells, 96- or 24-well plates were coated overnight at 4°C with anti-hIgG4 (1 μg/ml, BD Biosciences). Plates were washed with PBS and incubated for 2 hours at 37°C with 20 μg/ml Delta1-Fc or hIgG4 control, washed once again with PBS, before cells were plated for stimulation.

To examine the effect of coincident Delta1-Fc and T-cell receptor signalling on primary CD4^+ ^T-cells, 96- or 24-well plates were first coated overnight at 4°C with a mixture of capture antibodies consisting of anti-mouse IgG2a (1 μg/ml, BD Biosciences) and anti-hIgG_4 _(1 μg/ml, BD Biosciences). Plates were washed with PBS and incubated 2 hours at 37°C with a mixture of anti-CD3 (3 μg/ml, clone Hit3a, BD Biosciences) and 20 μg/ml Delta1-Fc or hIgG_4 _control (Sigma). Plates were again washed with PBS and purified CD4^+ ^T cells were seeded at 2 × 10^5^/well in the presence of soluble anti-CD28 (2 μg/ml, clone CD28.2, BD Bioscience).

### Inter-cell Notch signalling assay

This assay has been described previously [20]. Briefly, a CHO cell line (CHO-N2) was established which expressed both a full-length human Notch2 cDNA, and p10xCBF1-luc. 2 × 10^4 ^CHO-N2 cells were plated per well into 96-well plates, and left to adhere for 2 hours. Notch signalling was activated by plating an additional CHO line which stably expresses full-length human Delta1 (CHO-hDelta1) onto the CHO-N2 cells. Cells were co-cultured in this manner overnight, and luciferase activity determined as described.

### Reverse transcription and Quantitative PCR

RNA was isolated from cell samples using Rneasy kits (Qiagen). First strand cDNA was synthesised with SuperScriptII (Invitrogen) oligo-d(T) and random decamers (Invitrogen). Q-PCR was performed using SYBR green detection chemistry on the Roche Lightcycler system. Gene-specific quantification was achieved by comparing each cDNA to a serially-diluted standard plasmid containing the relevant amplicon. Relative Hes-1 expression levels were normalised to 18S rRNA housekeeping gene. Primer sequences used were as follows (5' to 3'): 18S rRNA, GTAACCCGTTGAACCCCATT and CCATCCAATCGGTAGTAGCG; Hes-1, CTCTCTTCCCTCCGGACTCT and GGCGCAATCCAATATGAAC.

## Authors' contributions

GM and GH conceived this study and wrote the manuscript. GM was experimentally involved in CHO, Jurkat and primary T-cell assays. GH was experimentally involved in the neuronal studies. GW carried out all the CHO and Jurkat assays and the primary T-cell experiments involving N1-IC transfection. YS and EB carried out the primary T-cell experiments involving Delta1-Fc stimulation. SP was experimentally involved in the neuronal studies. AL, BC and MT provided susbstantial intellectual input into all aspects of this study. All authors read and approved the final manuscript.
